# Long Non-Coding RNA in Gastric Cancer: Mechanisms and Clinical Implications for Drug Resistance

**DOI:** 10.3389/fonc.2022.841411

**Published:** 2022-01-28

**Authors:** Ying Liu, Xiang Ao, Yu Wang, Xiaoge Li, Jianxun Wang

**Affiliations:** ^1^Institute for Translational Medicine, The Affiliated Hospital of Qingdao University, Qingdao Medical College, Qingdao University, Qingdao, China; ^2^School of Basic Medical Sciences, Qingdao Medical College, Qingdao University, Qingdao, China

**Keywords:** long non-coding RNA, gastric cancer, drug resistance, biomarker, therapeutic target

## Abstract

Gastric cancer (GC) is the third leading cause of cancer-related deaths worldwide, with high recurrence and mortality rate. Chemotherapy, including 5-fluorouracil (5-FU), adriamycin (ADR), vincristine (VCR), paclitaxel (PTX), and platinum drugs, remains one of the fundamental methods of GC treatment and has efficiently improved patients’ prognosis. However, most patients eventually develop resistance to chemotherapeutic agents, leading to the failure of clinical treatment and patients’ death. Recent studies suggest that long non-coding RNAs (lncRNAs) are involved in the drug resistance of GC by modulating the expression of drug resistance-related genes *via* sponging microRNAs (miRNAs). Moreover, lncRNAs also play crucial roles in GC drug resistance *via* a variety of mechanisms, such as the regulation of the oncogenic signaling pathways, inhibition of apoptosis, induction of autophagy, modulation of cancer stem cells (CSCs), and promotion of the epithelial-to-mesenchymal transition (EMT) process. Some of lncRNAs exhibit great potential as diagnostic and prognostic biomarkers, as well as therapeutic targets for GC patients. Therefore, understanding the role of lncRNAs and their mechanisms in GC drug resistance may provide us with novel insights for developing strategies for individual diagnosis and therapy. In this review, we summarize the recent findings on the mechanisms underlying GC drug resistance regulated by lncRNAs. We also discuss the potential clinical applications of lncRNAs as biomarkers and therapeutic targets in GC.

## Introduction

Gastric cancer (GC) is one of the most serious malignant gastrointestinal neoplasms worldwide, representing the third leading cause of cancer-related mortality after lung cancer and liver cancer (1). According to the latest statistics from GLOBOCAN database, about more than one million new GC cases and approximately 769,000 deaths occurred in 2020 (2). Although the overall incidence of GC has shown a downward trend, it is still a terminal disease threatening human health and considerably affecting the quality of human lives. Currently, chemotherapy is still the best option for metastatic GC patients followed by surgical resection, and it can effectively prolong their five-year overall survival (OS) rate and quality of life. The commonly used chemotherapeutic drugs for GC treatment mainly include 5-fluorouracil (5-FU), adriamycin (ADR), vincristine (VCR), paclitaxel (PTX), and platinum drugs (3). At the beginning of chemotherapy, the efficiency of these drugs is remarkable and the prognosis is good. However, poor or even no response to chemotherapy is often observed in GC patients due to the development of drug resistance, which ultimately leads to the failure of clinical treatment and patients’ death (4). The underlying mechanisms of GC drug resistance are very complicated and still not fully understood. Therefore, in-depth research is of great importance for GC patients in the development of individual diagnosis and therapeutic strategy (5, 6).

Long non-coding RNAs (lncRNAs) are a large class of endogenous ncRNAs with a length of more than 200 nucleotides (7). In recent years, with the rapid development of bioinformatics analysis and the application of high-throughput sequencing technologies, a large number of lncRNAs has been found to occur widely and stably in eukaryotes, including those of plant, animal, and human being (8, 9). They are involved in various biological processes, such as proliferation, apoptosis, invasion, and metastasis, by regulating the timing and degree of gene expression *via* multiple mechanisms, including signal, decoy, scaffold, guide, and SINEUPs (9). In addition, lncRNAs also play crucial roles in the regulation of multiple pathological and physiological processes, such as embryonic development, gene expression regulation, and carcinogenesis, by acting as a competing endogenous RNA (ceRNA) to modulate the expression of specific microRNAs (miRNAs), thereby targeting the downstream genes of these miRNAs (10–12). Therefore, the dysregulation of lncRNAs is closely associated with a lot of diseases, including GC (13–16). In fact, the aberrant expression of lncRNAs has been observed in GC samples and cell lines (17, 18). A growing amount of evidence suggests that lncRNAs are involved in practically all aspects of GC progression, including drug resistance (19–21). Exosomes are a novel extracellular vesicles that play crucial roles in cancer progression by transferring multiple biologically active molecules, such as proteins, lipids, and lncRNAs. It has been shown that exosomes can mediate intracellular communication to promote drug resistance in cancer (22, 23). A large number of lncRNAs are found to be selectively enriched in exosomes and some of them also exhibit great clinical value as biomarkers and/or therapeutic targets for drug-resistant GC patients (6, 24).

In this review, we systematically summarize the characteristics of lncRNAs associated with drug resistance in GC, specifically exploring the underlying mechanisms by which lncRNAs produce the drug resistance. Additionally, we highlight their clinical applications as biomarkers and therapeutic targets for GC patients.

## Long Non-Coding RNAs and Chemotherapeutic Drugs in Gastric Cancer

Currently, the first-line chemotherapeutic drugs commonly used in clinic for GC patients include 5-FU, platinum drugs, ADR, PTX, and VCR, all of which have effectively improved the poor outcome of patients. However, the development of resistance to these drugs is still the principal limiting factor in achieving cures in GC patients. Drug resistance can be divided into single drug resistance and multidrug resistance (MDR) (25–27). In the following section, we will describe the lncRNAs involved in GC drug resistance (**Table 1**).

**Table 1:** LncRNA and chemotherapeutic drugs in GC.

Chemotherapeutic drug	lncRNAs	Alteration	Effect on chemotherapy	References
5-FU	MACC1-AS1; PVT1; HAGLR; FGD5-AS1; UCA1; EIF3J-DT; FEZF1-AS1; ANRIL; LINC00943; XLOC_006753	Up	Reduction	(3, 28–37)
	CRNDE; LEIGC; ADAMTS9-AS2;	Down	Enhancement	(19, 38, 39)
CDDP	MALAT1; PCAT-1; HOTTIP; CRNDE; MCM3AP-AS1; HMGA1P4; SNHG5; UCA1; LINC01572; HOSD-AS1; SNHG7; XIST; HOTAIR; DANCR; BANCR; PVT1; ROR; SNHG6; LOC101928316; FAM84B-AS; HCG11; FOXD1-AS1; GHET1; HCP5; ANRIL; ASB16-AS1; HULC; RP11-874J12.4; SNHG3; FGD5-AS1	Up	Reduction	(34, 39–67)
	HULC; CRAL; CASC2	Down	Enhancement	(68–70)
OXA	MALAT1; BLACAT1; DDX11-AS1; EIF3J-DT; LINC00200; LINC00641; NORAD; NEAT1	Up	Reduction	(32, 71–77)
	CRNDE	Down	Enhancement	(19)
ADR	ROR; CASC9; HOTAIR; UCA1; LINC01667; NEAT1; D63785; MURL	Up	Reduction	(78–85)
	GAS5	Down	Enhancement	(86)
PTX	CASC9; ZFAS1; HOTAIR; PVT1	Up	Reduction	(79, 87–89)
VCR	ROR; MURL	Up	Reduction	(78, 85)

### Long Non-Coding RNAs and 5-Fluorouracil

5-FU is an antimetabolite drug that has been widely applied for the treatment of different types of cancer in clinic, including GC (90). 5-FU is converted intracellularly to several active metabolites, including fluorodeoxyuridine monophosphate, fluorodeoxyuridine triphosphate, and fluorouridine triphosphate (91). These active metabolites can interfere in DNA replication by inhibiting thymidylate synthase (TS) activity and can also induce cytotoxicity by misincorporating its metabolites into RNA and DNA (92). However, its clinical application is still limited due to the resistance development of GC patients to this drug. Multiple lncRNAs have been found to be involved in GC resistance to 5-FU. Several oncogenic lncRNAs, such as metastasis associated in colon cancer 1-antisense 1 (MACC1-AS1), Plasmacytoma variant translocation 1 (PVT1), and HOXD antisense growth-associated long noncoding RNA (HAGLR), can promote the 5-FU resistance of GC cells. For instance, the upregulation of MACC1-AS1, induced by transforming growth factor β1 (TGF-β1), was shown to facilitate the fatty acid oxidation (FAO)-dependent resistance of GC cells to 5-FU by sponging miR-145-5p (93). In another study, Hu et al. revealed that HAGLR was upregulated in both GC tissues and cell lines. The overexpression of HAGLR significantly promoted the 5-FU resistance of GC cells by increasing the lactate dehydrogenase-A (*LDHA*) expression *via* sponging miR-338-3p. Consistent with this, the silencing of HAGLR sensitized GC cells to 5-FU (29). In addition, Du et al. showed that PVT1 promoted the drug resistance of GC cells to 5-FU through the activation of B-cell lymphoma-2 (Bcl-2) (28).

In contrast, several tumor suppressor lncRNAs can reverse 5-FU resistance in GC. For instance, Zhang et al. found that lncRNA colorectal neoplasia differentially expressed (CRNDE) was downregulated in 5-FU-resistant GC cells. The overexpression of CRNDE significantly enhanced the chemosensitivity of GC cells to 5-FU by increasing the expression of the long isoform of *PICALM* (encoding PICALML) by directly targeting serine/arginine-rich splicing factor 6 (SRSF6) (19). Furthermore, Han et al. revealed that lncRNA lower expression in gastric cancer (LEIGC) expressed at low levels in GC tissues. The overexpression of LEIGC significantly promoted the sensitivity of GC cells to 5-FU by inhibiting the epithelial-to-mesenchymal transition (EMT) process, whereas the knockdown of LEIGC showed the opposite effect (38).

### Long Non-Coding RNAs and Platinum Drugs

Platinum drugs, such as cisplatin (CDDP), oxaliplatin (OXA), and carboplatin, are widely used in the treatment of human cancers, including GC (94). These drugs represent a class of cell cycle non-specific chemotherapeutic agents that can directly insert platinum into DNA to generate monoaddutcts and DNA crosslinks, which block the unwinding of DNA double helix and inhibit DNA replication, finally leading to the necrosis or apoptosis of tumor cells (3). Moreover, the monoaddutcts formed by platinum drugs and DNA also can interact with several effector proteins to produce a series of abnormal signaling factors, thereby inducing cell death (95). However, the clinical success of platinum drugs is limited due to their severe, dose-limiting side effects and resistance to treatment. Multiple lncRNAs have been reported to be involved in the GC resistance to platinum drugs (3, 96)

Several oncogenic lncRNAs can promote the resistance of GC cells to platinum drugs. For instance, Pei et al. showed that lncRNA small nucleolar RNA host gene 7 (SNHG7) was upregulated in GC tissues and CDDP-resistant cells. The silencing of SNHG7 significantly enhanced the sensitivity of the CDDP-resistant cells. Mechanistically, SNHG7 conferred to resistance of GC cells to CDDP by downregulating the expression of *LDHA via* sponging miR-34a (49). In another study, Li et al. revealed that lncRNA prostate cancer-associated transcript 1 (PCAT-1) was highly expressed in CDDP-resistant GC tissues and cell lines. Similarly, PCAT-1 knockdown also enhanced the sensitivity of chemoresistant GC cells to CDDP. Mechanistically, PCAT-1 epigenetically silenced phosphatase and tensin homolog deleted on chromosome 10 (*PTEN*) by upregulating trimethylation of histone H3 lysine 27 (H3K27me3) *via* targeting enhancer of zeste homolog 2 (*EZH2*). Consistent with this, the knockdown of PTEN counteracted the PCAT-1 silencing-induced enhancement in the CDDP sensitivity of the chemoresistant GC cells (41). In addition, Luo et al. found that lncRNA EIF3J-DT was overexpressed in OXA-resistant GC tissues and cell lines. EIF3J-DT promoted the drug resistance of GC cells to OXA by increasing autophagy-related gene 14 (*ATG14*) expression *via* sponging miR188-3p (32).

In contrast, a number of tumor suppressor lncRNAs can reverse the platinum drugs resistance. For instance, lncRNA a disintegrin-like and metalloprotease with thrombospondin type I motif 9 antisense RNA 2 (ADAMTS9-AS2) was found to be downregulated in GC tissues and cell lines. Specifically, the overexpression of ADAMTS9-AS2 enhanced the sensitivity of chemoresistant GC cells to CDDP by increasing the *NACHT*, *LRR*, and PYD domains-containing protein 3 (*NLRP3*) expression *via* sponging miR-223-3p (39). Another study revealed that the expression lncRNA cisplatin resistance-associated lncRNA (CRAL) was decreased in CDDP-resistant GC cells. The overexpression of CRAL promoted the enhancement of CDDP sensitivity of GC cells by upregulating the expression of cylindromatosis *(CYLD) via* sponging miR-505 (69). Additionally, in another study, lncRNA cancer susceptibility candidate 2 (CASC2) overexpression enhanced the CDDP sensitivity of GC cells by sponging miR-19a, whereas CASC2 knockdown reversed the response of GC cells to CDDP. Consistent with this, miR-19a overexpression counteracted the CASC2 overexpression-induced enhancement in the CDDP sensitivity of GC cel (70).

### Long Non-Coding RNAs and Adriamycin

ADR, also known as doxorubicin (DOX), is one of the most commonly prescribed and time-tested chemotherapeutic drugs (97). It can produce free radical to induce the impairment of DNA and the cell membrane, thereby resulting in tumor cell death. Moreover, ADR suppresses the biosynthesis of macromolecules by interacting with DNA *via* intercalation, thereby inhibiting DNA topoisomerase II, and leading to the stagnation of DNA replication (98). Several oncogenic lncRNAs, such as lncR-D63785, urothelial cancer-associated 1 (UCA1), and nuclear paraspeckle assembly transcript 1 (NEAT1), have been reported to promote ADR resistance in GC (81, 83, 84). For instance, in our previous work, we found that lncR-D63785 was highly expressed in both GC tissues and cell lines. The overexpression of lncR-D63785 conferred the ADR resistance of GC cells by increasing myocyte enhancer factor 2D *(MEF2D)* expression *via* sponging miR-422 (84). In another study, Shang showed that UCA1 was significantly upregulated in GC tissues and cells. The silencing of UCA1 could reverse the ADR resistance of GC cells and promote apoptosis induced by ADR by increasing cleaved Poly(ADP-ribose) polymerase (*PARP*) expression and decreasing *Bcl-2* expression (81). Similarly, Zhang et al. showed that NEAT1 knockdown in ADR-resistant GC cells could enhance the sensitivity of GC cells to ADR and promote apoptosis induced by AD (83). In addition, another two lncRNAs, regulator of reprogramming (ROR) and MDR-related and upregulated lncRNA (MURL), were also found to promote ADR resistance in GC cells *via* regulating the expression of various genes (85, 99).

### Long Non-Coding RNAs and Paclitaxel

PTX is a tricyclic diterpenoid compound produced from *Taxus brevifolia* and is considered to be one of the most successful natural chemotherapeutic agents available (100). As an antimitotic agent, PTX facilitates the assembly of tubulin into microtubules and the lengthening of the tubulin polymer, leading to the inhibition of microtubule depolymerization. The enhancement of microtubule stability interferes with microtubules’ polymerization dynamics, leading to G2/M cell cycle arrest of tumor cells (101, 102). Several oncogenic lncRNAs have been shown to be responsible for the development of PTX resistance in GC. For instance, Shang et al. found that lncRNA cancer susceptibility candidate 9 (CASC9) was significantly upregulated in both GC tissues and cells. The silencing of CASC9 in drug-resistant GC cells restored the sensitivity of cells to PTX *via* downregulating the expression of multidrug resistance protein 1 (*MDR1*) (79). Moreover, An et al. revealed that lncRNA ZNFX1 antisense RNA 1 (ZFAS1) was highly expressed in GC tissues. The knockdown of ZFAS1 enhanced the sensitivity of GC cells to PTX by decreasing β-catenin expression. Consistent with this, the overexpression of β-catenin reversed the ZFAS1 knockdown-induced resistance of GC cells to PTX (87). Wang et al. showed that lncRNA HOX transcript antisense RNA (HOTAIR) was upregulated in GC tissues. The overexpression of HOTAIR enhanced the PTX and ADR resistance in GC cells by upregulating glypican-5 (*GPC5*) and protein tyrosine phosphatase non-receptor type 14 (*PTPN14*) expression *via* sponging miR-217 (88). In another study, Hu et al. demonstrated that the expression of lncRNA metastasis-associated lung adenocarcinoma transcript 1 (MALAT1) was upregulated in PTX-resistant GC cells and that the silencing of MALAT1 enhanced the sensitivity of GC cells to PTX. Mechanistically, MALAT1 acted as a ceRNA to decrease ATG12 expression by sponging miR-23b-3p, leading to the enhancement of PTX resistance in GC cells (103).

### Long Non-Coding RNAs and Vincristine

VCR is a vinca alkaloid that is often used in combination with other chemotherapeutic agents to treat a variety of cancers, including GC (104). Similar with PTX, VCR is also an antimitotic agent that blocks tubulin polymerization and disables spindle formation. This inhibition causes the mitosis of tumor cells to arrest at metaphase, particularly during the M and S phases (105). In addition, VCR also can interfere with the synthesis of nucleic acids and proteins by blocking glutamic acid utilization (106). Several lncRNAs have been reported to be involved in the resistance of GC cells to VCR. For instance, the high expression of oncogenic lncRNA ROR was associated with the increased MDR of GC patients. The silencing of ROR promoted the apoptosis of drug-resistant GC cells in response to VCR and ADR treatment by decreasing multidrug resistance-associated protein 1 *(MRP1)* expression (78). In Wang et al.’s study, lncRNA MRUL was found to be upregulated in the VCR-resistant GC cells. The knockdown of MRUL promoted apoptosis and decreased VCR release in the VCR-resistant GC cells by upregulating *MRP1* expression (85).

## Mechanisms of Long Non-Coding RNAs in Gastric Cancer Drug Resistance

**Figure 1:**
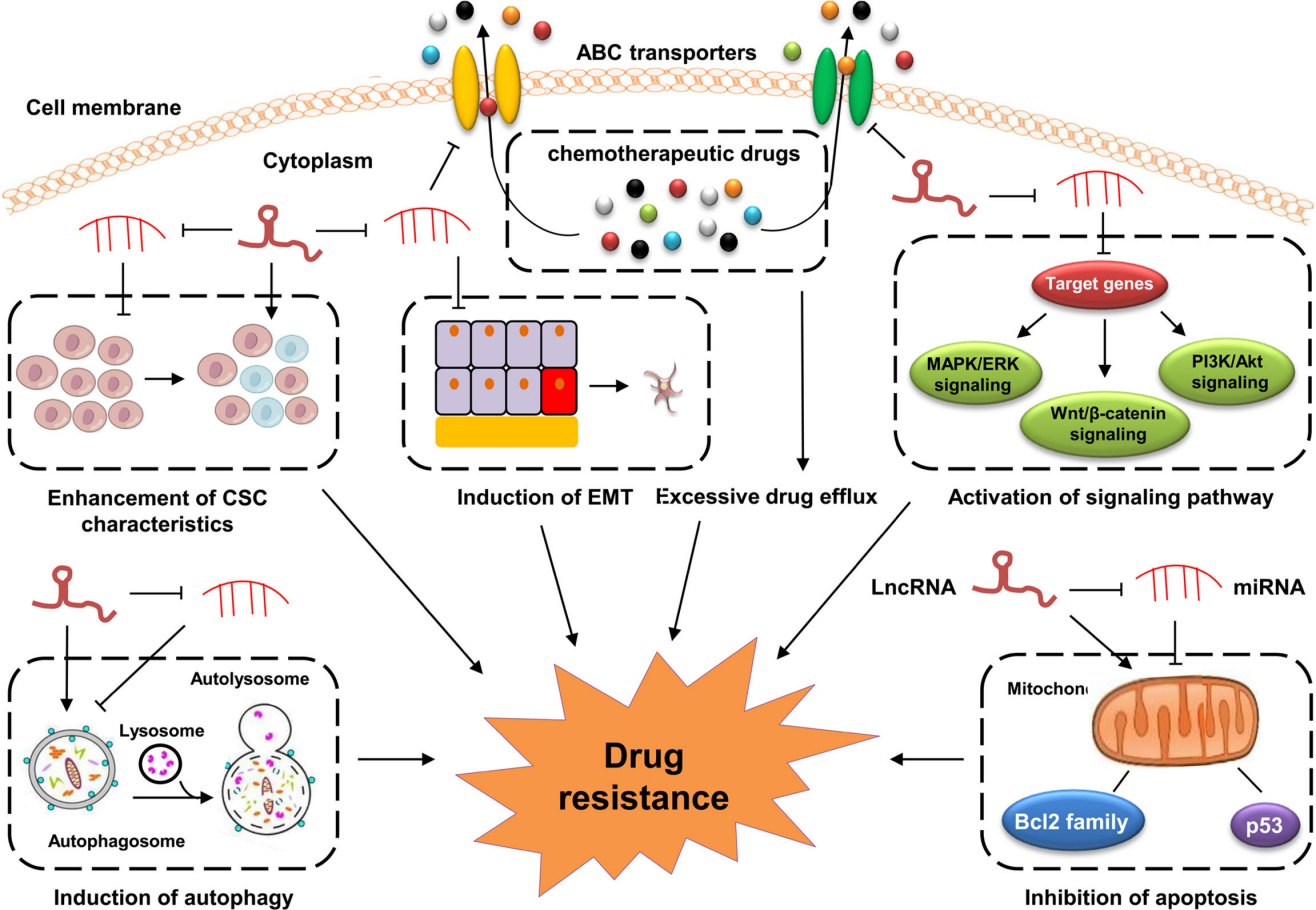
Mechanisms of lncRNAs in GC drug resistance. Dysregulated lncRNAs are involved in GC drug resistance by directly binding to related proteins or sponging miRNAs to altering the expression of downstream target genes involved in cell apoptosis, drug efflux system, EMT, CSCs and drug resistance-related signaling pathways.

The underlying mechanisms of GC drug resistance are very complex and still not fully understood. LncRNAs have been shown to play crucial roles in the development of GC drug resistance, indicating their great potential in precisely evaluating the sensitivity of cancer cells to chemotherapy in GC. LncRNAs regulate the development of drug resistance in GC *via* various mechanisms, such as the regulation of the oncogenic signaling pathways, inhibition of apoptosis, induction of autophagy, modulation of CSCs, and promotion of the EMT process (**Figure 1**).

### Long Non-Coding RNAs Target Signaling Pathways in Gastric Cancer Drug Resistance

**Figure 2:**
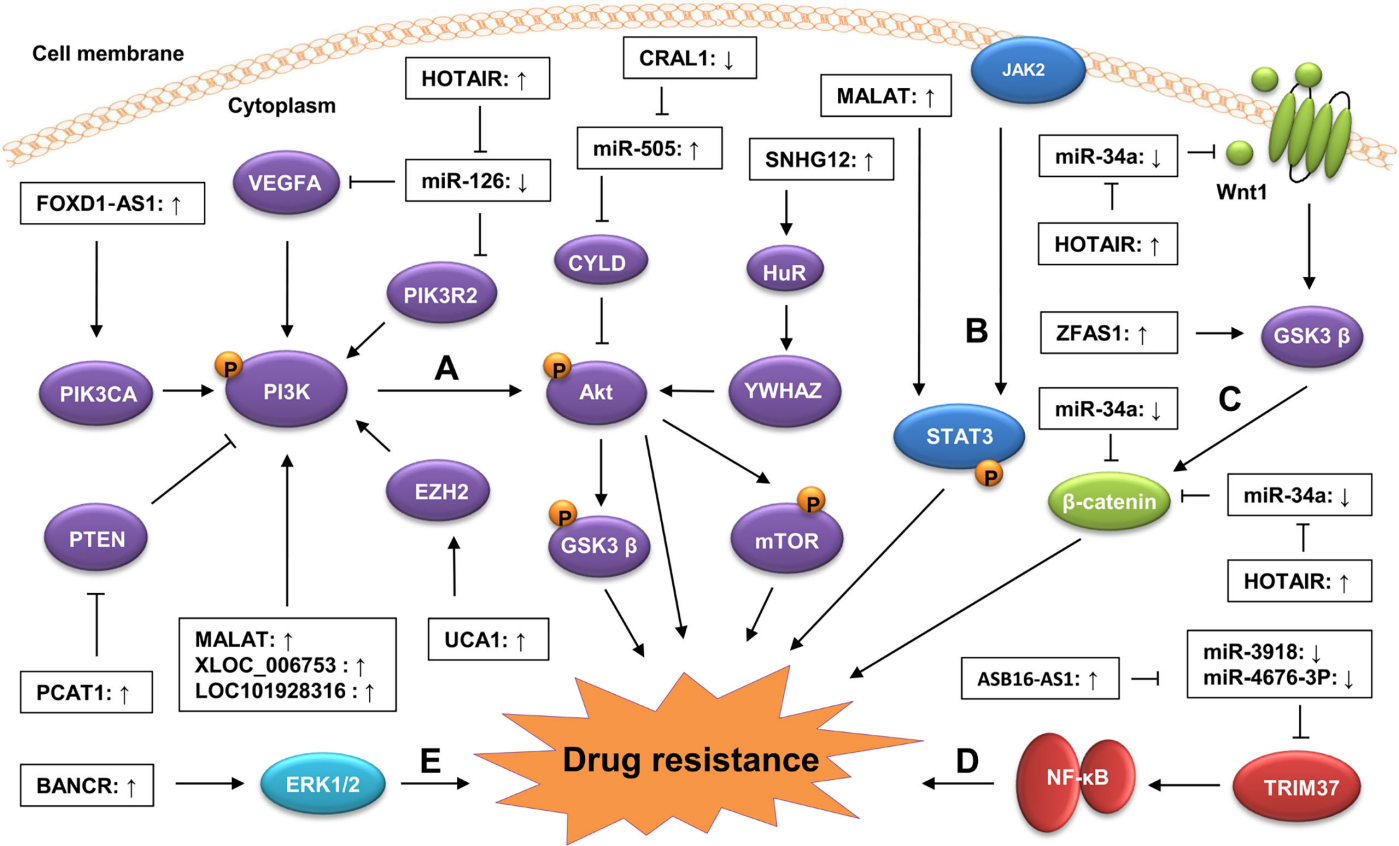
Regulation of lncRNAs on signaling pathways in GC drug resistance. Dysregulated lncRNAs contribute to GC drug resistance by targeting oncogenic signaling pathways, including the PI3K/AKT **(A)**, STAT3 **(B)**, Wnt/β-catenin **(C)**, NF-kB **(D)**, and **(E)** MAPK/ERK signaling pathways. Generally, the dysregulated lncRNAs can activate oncogenic signaling pathways by modulating the expression of key components in these signaling pathways *via* sponging miRNAs. They also can influence these signaling pathways by altering the modification of their key components *via* regulating the activity of upstream regulators.

An increasing number of studies have shown that lncRNAs regulate GC drug resistance by targeting cancer-related signaling pathways, such as the phosphatidylinositol 3-kinase (PI3K)/AKT, Wnt/β−catenin, and mitogen-activated protein kinase/extracellular signal regulated kinase (MAPK/ERK) signaling pathways (46, 53, 107, 108) (**Figure 2**). These lncRNAs can change the sensitivity of GC cells to chemotherapy drugs by altering the activity or expression of some key components in these signaling pathways.

The PI3K/AKT signaling pathway is a carcinogenic pathway involved in a variety of physiological processes, including proliferation, apoptosis, differentiation, migration, and invasion (109). The aberrant activation of this pathway is closely associated with many aspects of cancer progression, including drug resistance (110, 111). Several lncRNAs have been found to be involved in GC drug resistance by modulating the activity of the PI3K/AKT signaling pathway. For instance, Dai et al. revealed that lncRNA MALAT1 was highly expressed in GC tissues and that its upregulation enhanced the resistance of GC cells to CDDP. Consistent with this, the knockdown of MALAT1 reduced the inhibition of MALAT1 on the apoptosis of the CDDP-resistant GC cells. Mechanistically, MALAT1 activated the PI3K/AKT signaling pathway by upregulating the level of phosphorylated PI3K (p-PI3K) and p-AKT, leading to the enhancement of the CDDP resistance of GC cells (40). Zeng et al. found that lncRNA XLOC_006753 promoted the resistance of GC cells to 5-FU and CDDP by activating the PI3K/AKT signaling pathway. The silencing of XLOC_006753 in MDR GC cells significantly decreased the levels of PI3K, p-AKT, and phosphorylated mechanistic target of rapamycin (p-mTOR) (36). Moreover, Wu et al. demonstrated that the high expression of lncRNA Forkhead Box D1-antisense 1 (FOXD1‐AS1) increased the resistance of GC cells to CDDP by activating the PI3K/AKT signaling pathway *via* the upregulation of *PIK3CA* (60). PTEN is a well-known negative regulator of the PI3K/AKT signaling pathway (112, 113). Li et al. showed that PCAT-1 could downregulate *PTEN* expression by targeting EZH2 in CDDP-resistant GC cells, indicating that PCAT-1 might contribute to the resistance of GC cells to CDDP by activating the PI3K/AKT signaling pathway *via* downregulating *PTEN* expression (41).

The Wnt/β−catenin signaling pathway is a conserved cancer-related pathway participating in cancer progression by promoting CSC renewal, proliferation, and differentiation, thereby playing crucial roles in carcinogenesis and therapy response (114, 115). The aberrant activation of the Wnt/β−catenin pathway contributes to the development of drug resistance in a variety of cancers, including GC (116, 117). Several lncRNAs have been reported to be involved in GC drug resistance by regulating the expression of key components in the Wnt/β-catenin signaling pathway. For instance, Xu et al. showed that lncRNA ZFAS1 was highly expressed in GC tissues. The knockdown of ZFAS1 enhanced the sensitivity of GC cells to PTX and cis-platinum by inactivating the Wnt/β-catenin signaling pathway *via* downregulating β-catenin and phosphorylated glycogen synthase kinase-3β (p-GSK3β) level and increasing naked cuticle homolog 2 *(NKD2)* expression. Consistent with this, the overexpression of β-catenin reversed the inhibition of the GC drug resistance induced by the ZFAS1 knockdown (87). In another study, Cheng et al. revealed that lncRNA HOTAIR was upregulated in both GC tissues and cell lines. The knockdown of HOTAIR reduced the resistance of GC cells to CDDP. Mechanistically, HOTAIR activated the Wnt/β-catenin signaling pathway by upregulating the level of Wnt1 and β-catenin *via* sponging miR-34a in GC cells, resulting in the enhancement of CDDP resistance (107). In addition, some lncRNAs, such as small nucleolar RNA hostgene 11 (SNHG11), HOXC cluster antisense RNA 1 (HOXC-AS1), and DLGAP1 antisense RNA 2 (DLGAP1-AS2), have also been found to regulate the expression of the key components in the Wnt/β-catenin signaling pathway during GC progression (20, 118–120), indicating that they may also influence the development of drug resistance in GC by targeting the Wnt/β-catenin pathway.

The MAPK/ERK signaling pathway is a classical oncogenic pathway, and its dysregulation has been shown to be closely associated with the development of drug resistance in GC (121, 122). Particularly, Miao et al. found that the overexpression of lncRNA BRAF-activated non-coding RNA (BANCR) enhanced the resistance of GC cells to CDDP by activating the MAPK/ERK signaling pathway *via* upregulating the level of p-ERK1/2. Consistent with this, Ly3214996 (an ERK inhibitor) treatment reversed the BANCR overexpression-induced CDDP resistance of GC cells by decreasing the p-ERK1/2 level (53). In addition, lncRNAs can also regulate GC drug resistance by targeting the nuclear factor-kappaB (NF-κB) signaling pathway. Fu et al. revealed that lncRNA ASB16 antisense RNA 1 (ASB16-AS1) promoted the CDDP resistance of GC cells by activating the NF-κB signaling pathway. Mechanistically, the overexpression of ASB16-AS1 upregulated the level of NF-κB pathway-related proteins (p-IKK, p-IκB and Nuclear p65) by increasing tripartite motif-containing 37 *(TRIM37)* expression *via* sponging miR-3918 and miR4676-3p, leading to the enhancement of CDDP resistance in GC (63). Collectively, these findings suggest that targeting the oncogenic signaling pathways is a common mechanism for lncRNAs in the development of GC drug resistance. Thus, in-depth studies on the underlying mechanisms of lncRNAs in the regulation of the GC signaling pathway may provide novel insights into the development of therapeutic strategies against GC drug resistance.

### Long Non-Coding RNAs Regulate Cell Apoptosis in Gastric Cancer Drug Resistance

An important function of most chemotherapeutic drugs is to promote the effective elimination of cancer cells by inducing apoptosis (123, 124). Therefore, the dysregulation of apoptosis is one of the main factors resulting in the occurrence of drug resistance. An increasing amount of evidence has shown that lncRNAs participate in the development of GC drug resistance by regulating apoptotic proteins or related pathways (65, 74, 125).

Bcl-2 family proteins are key regulators of the mitochondrial apoptotic pathway and are divided into two major groups: anti-apoptotic (e.g., Bcl-2, Mcl-1, and Bcl-xl) and pro-apoptotic (e.g., Bax, Bim, and Bak) (126, 127). Li et al. showed that lncRNA small nucleolar RNA hostgene 5 (SNHG5) was upregulated in CDDP-resistant GC tissues and cell lines. Specifically, SNHG5 overexpression in GC cells reduced the CDDP cytotoxicity and promoted cell apoptosis by upregulating Bax and downregulating Bcl-2 level. Consistent with this, the silencing of SNHG5 promoted the apoptosis of the CDDP-resistant GC cells (45). Qiao et al. revealed that lncRNA HMGA1P4 overexpression inhibited apoptosis of the CDDP-resistant GC cells by decreasing Bcl-2 and caspase3 expression and increasing Bax expression (37). Moreover, Fang et al. found that lncRNA UCA1 was highly expressed in GC tissues. The overexpression of UCA1 increased the MDR of GC cells to ADR, DDP, and 5-FU and decreased the cell apoptosis induced by ADR by upregulating Bcl-2 and downregulating caspase3 *via* sponging miR-27b. Consistent with this, UCA1 knockdown decreased the MDR of GC cells, and increased the ADR-induced cell apoptosis (31). Zhang et al. demonstrated that lncRNA gastric carcinoma high expressed transcript 1 (GHET1) overexpression significantly enhanced the resistance of GC cells to CDDP and inhibited cell apoptosis by downregulating Bax level and upregulating Bcl-2, MDR1 and MRP1 level. Conversely, the knockdown of GHET1 decreased MDR and promoted apoptosis in CDDP-resistant GC cells (61). Moreover, Zong et al. found that lncRNA LINC00162 overexpression increased the sensitivity of GC cells to 5-aza-2’-deoxycytidine and cell apoptosis by interacting with heterogeneous nuclear ribonucleoprotein H1 (HNRNPH1) and downregulating Bcl-XL level (128).

In addition, Cheng et al. showed that the overexpression of UCA1 could inhibit the apoptosis induced by CDDP in GC cells by upregulating cytochrome P450 1B1 enzyme *(CYP1B1)* expression *via* sponging miR-513a-3p, which lead to the enhancement of the CDDP resistance of GC cells (129). In another study, the overexpression of lncRNA differentiation antagonizing non-protein coding RNA (DANCR) was found to increase survival and decrease apoptosis in CDDP-resistant GC cells by upregulating the expression of *MDR1* and *MRP1* (52). Moreover, Wang et al. revealed that lncRNA CRAL overexpression promoted CDDP-induced apoptosis in GC cells by upregulating *CYLD* expression *via* sponging miR-505 (69). Zhang et al. demonstrated that lncRNA FAM84B-AS promoted the resistance of GC cells to platinum drugs by inhibiting the apoptotic pathways. Mechanistically, FAM84B-AS knockdown significantly increased the level of Bax, caspase3, caspase7, and caspase9 and then decreased the level of Bcl-2 and Bcl-xl by upregulating *FAM84B* expression to further inhibit the GC cell apoptosis (58). Taken together, these findings demonstrate that lncRNAs can act as oncogenes to contribute to drug resistance by suppressing cell apoptosis in GC, at least partially due to their modulation on the expression of apoptotic proteins. However, the exact mechanisms are still not fully elucidated. Therefore, in-depth studies are required to further understand the detailed mechanism of lncRNAs in GC drug resistance, which may provide new insights for the development of lncRNA-based therapeutics strategies for patients against drug resistance.

### Long Non-Coding RNAs Alter Drug Efflux In Gastric Cancer Drug Resistance

Excessive drug efflux mediated by multiple cell membrane transporter proteins, such as ATP-binding cassette (ABC) proteins, is one of the main mechanisms of drug resistance in cancer cells (130). It has been reported that the overexpression of ABC proteins could enhance the efflux of anticancer drugs from cancer cells, thereby reducing their efficacy and leading to the resistance of cancer cells to these anticancer drugs (131). Currently, at least three members of ABC family have been shown to be closely associated with MDR, particularly MDR1 (also known as ABCB1 and P-glycoprotein), MRP (also known as ABCC1), and breast cancer resistant protein (BCRP) (also known as ABCG2) (132).

**Figure 3:**
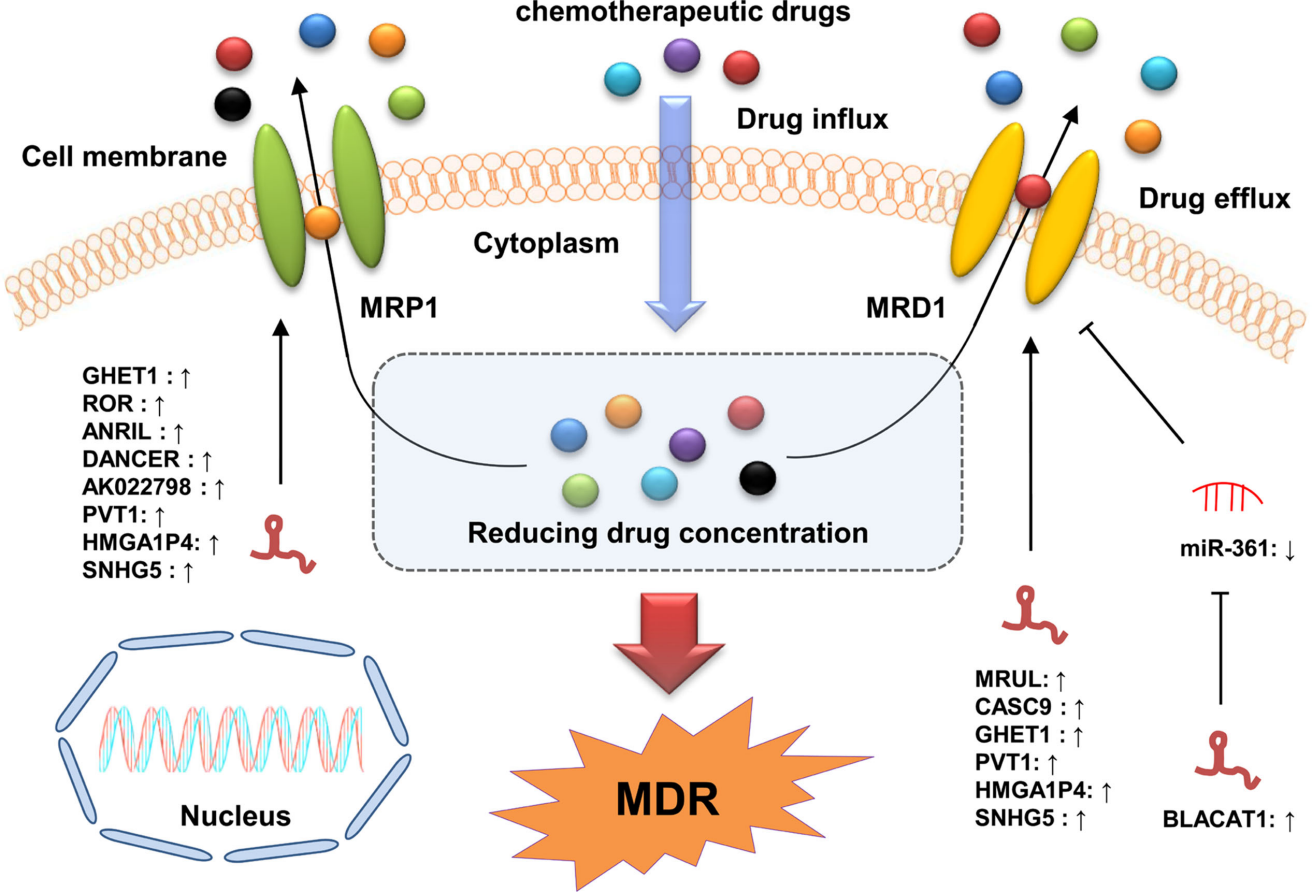
LncRNAs participate in GC drug resistance by regulating MDR-related genes. The ABC transporters export chemotherapeutic drugs out of GC cells, resulting in chemotherapy resistance with decreased concentrations of the drugs intracellularly. LncRNAs can modulate MDR by increasing the expression of MDR-related genes (*MDR1* and *MRP1*) *via* directly binding to specific miRNAs or proteins.

An accumulating number of studies have shown that lncRNAs participate in the regulation of excessive drug efflux in GC cells by targeting ABC proteins (**Figure 3**). MDR1 was the first-identified ABC protein associated with MDR (133). Several lncRNAs, such as bladder cancer-associated transcript 1 (BLACAT1), MRUL, PVT1, and CASC9, have been reported to regulate the development of MDR in GC by directly or indirectly targeting *MDR1*. For instance, Xu et al. found that lncRNA BLACAT1 was upregulated in OXA-resistant GC tissues and cell lines and that its overexpression promoted the OXA resistance of these GC cells by upregulating *MDR1* expression *via* sequestering miR-361 (72). Wang et al. revealed that lncRNA MRUL was highly expressed in MDR GC cells with the knockdown of MRUL enhancing the sensitivity of these cells to ADR and VCR by downregulating *MDR1* expression (85). Shang et al. showed that lncRNA CASC9 knockdown restored the sensitivity of MDR GC cells to PTX and ADR by decreasing *MDR1* expression (79). In addition, Zhang et al. demonstrated that the upregulation of lncRNA GHET1 facilitated the development of MDR in GC cells by upregulating MDR1, Bcl-2, and MRP1 level and downregulating Bax level (61). Taken together, these findings indicate that lncRNAs can participate in the regulation of drug resistance in GC cells by altering the drug efflux. Further investigations on the underlying mechanisms of lncRNAs in drug efflux regulation is of great importance in helping GC patients overcome drug resistance.

### Long Non-Coding RNAs and Epithelial-To-Mesenchymal Transition in Gastric Cancer Drug Resistance

EMT is a process by which epithelial cells lose their junctions and polarity, and gain migratory and invasive characteristics, producing a fibroblast-like phenotype (134, 135). The aberrant activation of the EMT process is closely associated with GC drug resistance (136). An increasing amount of evidence has shown that lncRNAs are involved in GC drug resistance by modulating the EMT process. For instance, Mao et al. revealed that lncRNA HOXA transcript at the distal tip (HOTTIP) was upregulated in CDDP-resistant GC cells. The knockdown of HOTTIP promoted the EMT progress by upregulating the level of the epithelial markers (E-cadherin and ZO-1) and downregulating the level of the mesenchymal markers (N-cadherin, vimentin, ZEB-1, and Twist), leading to the enhancement of MDR in GC cells (42). In another study, Han et al. found that lncRNA LEIGC was downregulated in GC tissues with its overexpression enhancing the sensitivity of GC cells to 5-FU by suppressing the EMT process (38). Moreover, Jia et al. demonstrated that lncRNA HOTAIR was highly expressed in GC tissues. The overexpression of HOTAIR significantly promoted the EMT process by increasing the level of N‐cadherin and vimentin and decreasing E‐cadherin level in GC cells that were subject to treatment *via* chemo‐therapies (80). In addition, some lncRNAs, such as metastasis-associated lung adenocarcinoma transcript-1 (MALAT-1), microvascular invasion in hepatocellular carcinoma (MVIH), NEAT1, and gastric cancer associated transcript 3 (GACAT3), have also been found to modulate the expression of EMT markers during GC progression (16, 137–140), indicating that these lncRNAs may also be involved in the development of drug resistance in GC by regulating the EMT process. EMT is a core link of metastatic process, which is closely associated with cancer recurrence and drug resistance. Therefore, dysregulated lncRNAs are likely to play a positive role in the development of GC drug resistance by aberrantly activating the EMT process. These findings support the hypothesis that lncRNAs are key regulators in GC drug resistance, but the molecular mechanisms still need further investigation.

### Long Non-Coding RNAs Influence Gastric Cancer Drug Resistance by Regulating Cancer Stem Cell Characteristics

CSCs are a unique subpopulation of cancer cells within a tumor with high tumorigenic potency and have been recognized as the main cause of the chemotherapeutic resistance, metastasis, and recurrence of cancer (141, 142). An accumulating number of studies have shown that lncRNAs can participate in the regulation of GC drug resistance by regulating the characteristics of CSC. For instance, Sun et al. showed that lncRNA small nucleolar RNA hostgene 3 (SNHG3) upregulation was positively associated with the CDDP resistance and stemness of GC cells, whereas SNHG3 downregulation inhibited the stem cell-like properties of these GC cells. Mechanistically, SNHG3 significantly enhanced the stemness of GC cells by increasing ADP-ribosylation factor-like protein 2 (*ARL2*) expression *via* sponging miR-3619-5p, leading to the enhancement of GC CDDP resistance (66). Fu et al. revealed that lncRNA ASB16 antisense RNA 1 (ASB16-AS1) was highly expressed in GC tissues. Specifically, the overexpression of ASB16-AS1 strengthened the stem cell-like features of GC cells by upregulating *TRIM37* expression *via* sponging miR-3918 and miR-4676-3p, resulting in the enhancement of CDDP resistance in GC (63). Wang et al. found that lncRNA ROR was highly expressed in CD133+ gastric CSCs (GCSCs). The overexpression of ROR promoted the pluripotent state of GCSCs by upregulating several key stemness transcriptional factors, such as octamer-binding transcription factor 4 (*OCT4*), SRY-box transcription factor 2 (*SOX2*), and *NANOG* (143). Furthermore, lncRNAs MALAT1 and testis-associated highly-conserved oncogenic long non-coding RNA (THOR) have been shown to increase the stemness of GC cells by enhancing *SOX2* and *SOX9* mRNA stability, respectively (144–146). In addition, the upregulation of lncRNAs histocompatibility leukocyte antigen complex P5 (HCP5) and MACC1-AS1 induced *via* mesenchymal stem cell (MSC) co-culturing was found to enhance the stemness and drug-resistance of GC cells by droving FAO (93, 147).

### Long Non-Coding RNAs and Autophagy in Gastric Cancer Drug Resistance

**Figure 4:**
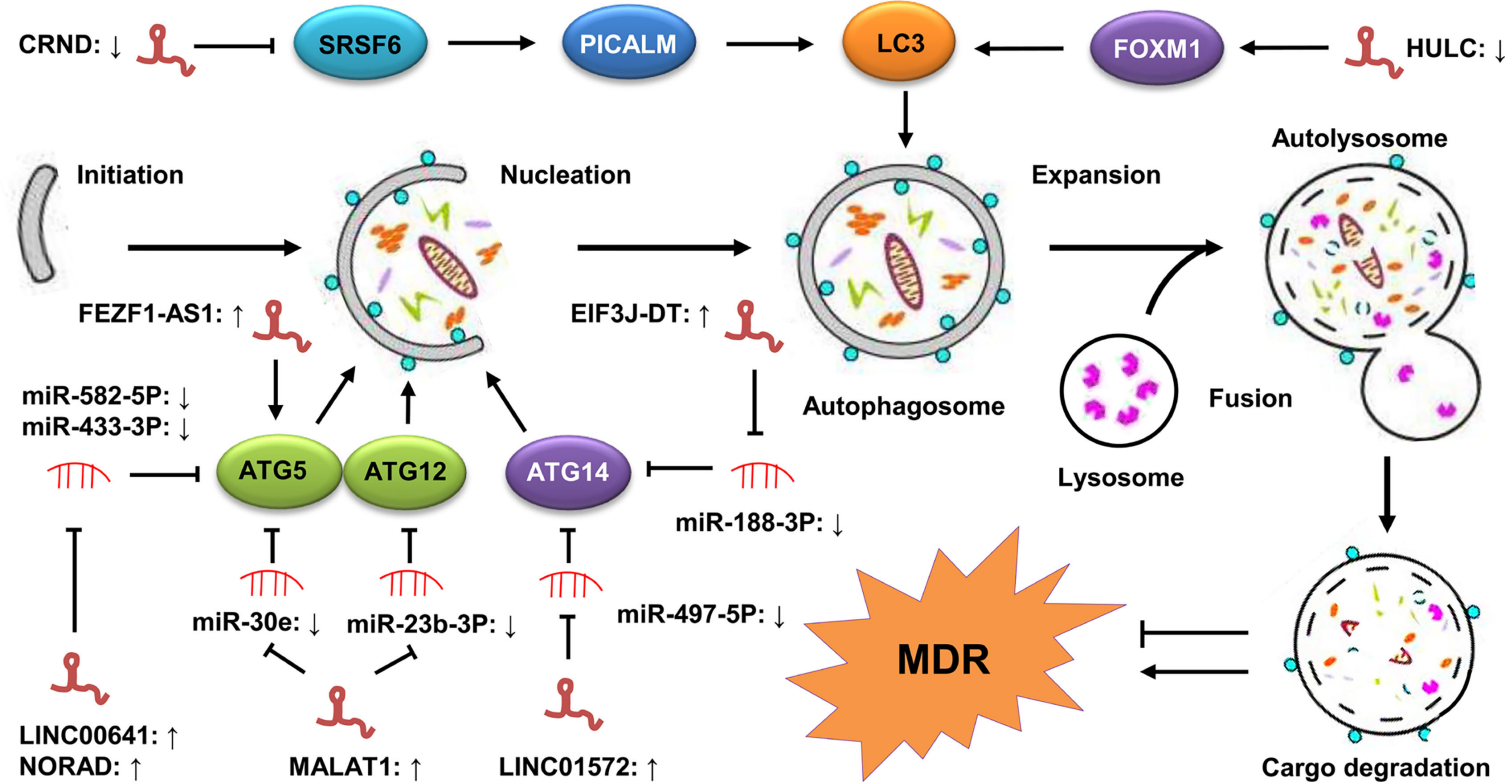
LncRNAs regulate autophagy-mediated GC drug resistance. The process of autophagy contains five steps, including initiation, vesicle nucleation, vesicle expansion, vesicle fusion, and cargo degradation. These steps are tightly regulated by a series of ATGs, including ATG5, ATG12, ATG14 and LC3. LncRNAs are involved in autophagy-mediated MDR of GC cells by modulating the expression of these ATGs *via* sponging miRNAs or directly targeting them.

Autophagy is a complex, highly conserved self-metabolic process that is essential for maintaining cellular homeostasis during stress conditions, such as hypoxia and nutrient deprivation (148, 149). It has been reported that autophagy plays dual roles in GC drug resistance. On the one hand, it can promote drug resistance by protecting GC cells from the cytotoxicity of chemotherapeutic agents. On the other hand, it can reverse drug resistance through facilitating apoptosis and/or inhibiting EMT (150). Therefore, targeting autophagy may be one of the crucial therapeutic strategies against GC drug resistance. Furthermore, an increasing number of studies have shown that lncRNAs are involved in GC drug resistance by targeting autophagy (68, 103) (**Figure 4**).

Zhang et al. showed that the expression of lncRNA CRNDE was downregulated in CDDP-resistant GC cells. The low level of CRNDE promoted the MDR of GC cells to 5-FU and OXA by upregulating autophagy flux in GC. Mechanistically, CRNDE reduced the alternative splicing of *PICALM* by decreasing the protein stability of SRSF6, leading to the enhancement of autophagy-mediated drug resistance (19). In another study, Wang et al. found that lncRNA non-coding RNA activated by DNA damage (NORAD) was highly expressed in OXA-resistant GC tissues, with its upregulation in the OXA-resistant GC cells promoting the autophagy flux by stabilizing the autophagy related gene 5 (ATG5)-ATG12 complex *via* sponging miR-433-3p, leading to the enhancement of CDDP resistance (76). Moreover, Luo et al. revealed that lncRNA EIF3J-DT was upregulated in drug-resistant GC cells. The high expression of EIF3J-DT activated autophagy and induced drug resistance in these GC cells by upregulating *ATG14* expression *via* sequestering miR188-3p (32). In addition, some lncRNAs, such as FEZF1 antisense RNA 1 (FEZF1-AS1), LINC00641, and MALAT1, have also been found to mediate drug resistance in GC by activating autophagy (33, 75, 103). Collectively, these findings suggest that the lncRNA regulation of autophagy is an important mechanism in the development of GC drug resistance. However, how lncRNAs participate in GC drug resistance by modulating autophagy is still not fully understood. Accordingly, in-depth studies are required to elucidate its exact mechanisms.

## Clinical Implication of Drug Resistance-Related Long Non-Coding RNAs in Gastric Cancer

### Long Non-Coding RNAs as Diagnostic and Prognostic Biomarkers

The recurrence and mortality rates of GC are still high due to the lack of efficient approaches for its early diagnosis and prognostic assessment in clinical practice. Currently, some conventional biomarkers, such as carcinoembryonic antigen (CEA), carbohydrate antigen 19-9 (CA19-9), and cancer antigen 72-4 (CA72-4), have been used in clinic, but the low diagnostic accuracies of these biomarkers limit their further application (151, 152). Thus, the screening and identification of valuable molecular biomarkers with high specificity and sensitivity is very essential for GC patients, particularly those with a poor response to chemotherapy, to obtain timely and effective treatment.

In recent years, a large number of studies have shown the key role of drug resistance-related lncRNAs in the pathogenesis of GC. Additionally, the differential expression of these lncRNAs endows them with great potential as biomarkers for the diagnosis, prognosis and treatment of GC patients (**Table 2**). For instance, the expression of lncRNA SNHG5 was significantly downregulated in the serum and tissues of GC patients before operation as well as those with poor prognosis, and its downregulation was closely associated with drinking history and Tumor-Node-Metastasis (TNM) stages in these patients, indicating that SNHG5 is a survival predictor of short-term adverse events in GC patients (155). In another study, the overexpression of SNHG5 decreased CDDP cytotoxicity and inhibited apoptosis in GC cells (45). This data suggests that SNHG5 processes a high diagnostic value in prognosis prediction and is a promising biomarker for the early diagnosis of GC. In another study, lncRNA PVT1 was upregulated in both the tissues and cells of CDDP‐resistant patients. This overexpression of PVT1 promoted the apoptosis induced by CDDP in GC cells by upregulating the expression of MDR‐related genes, such as *MDR1*, *mTOR*, and *MRP* (156). Furthermore, the upregulation of PVT1 was closely associated with late tumor stage (III–IV), lymph node metastasis, and OS/disease-free survival (DFS) (153). These findings provide evidence that PVT1 can be used as a candidate biomarker for the diagnosis and personalized therapeutic treatment of GC patients. Besides, lncRNA NORAD was found to be highly expressed in OXA-resistant GC tissues. The overexpression of NORAD enhanced the autophagy flux in OXA-resistant GC cells by stabilizing the ATG12 complex *via* sponging miR-433-3p. This data suggests that NORAD can serve as a clinically useful biomarker for predicting OXA resistance in GC. In addition, some drug resistance-related lncRNAs, such as UCA1, DDX11 antisense RNA 1 (DDX11-AS1), and XLOC_006753, have also exhibited great potential as prognostic biomarkers for GC patients (36, 73, 81). However, the potential value of lncRNAs as biomarkers for GC patients needed be further confirmed *via* larger population-based studies.

**Table 2:** Drug resistance-related lncRNAs as diagnostic and prognostic biomarkers in GC.

LncRNAs	Alteration	Potential values	References
CRAL	Down	Low level of CRAL predicts poor response to chemotherapy.	(69)
UCA1	Up	High UCA1 expression predicts poorer survival and poor response to chemotherapy.	(81)
LINC00641	Up	High level of UCA1 predicts poorer survival and poor response to chemotherapy.	(75)
NORAD	Up	High NORAD expression predicts poor response to chemotherapy.	(76)
DDX11-AS1	Up	High level of D DX11-AS1 predicts poor response to chemotherapy.	(73)
PVT1	Up	High PVT1 expression predicts expression predicts poor prognosis and poor response to chemotherapy	(153, 154).
MCM3AP-AS1	Up	High level of MCM3AP-AS1 predicts poor response to chemotherapy.	(44)
XLOC_006753	Up	High level of XLOC_006753 predicts poorer survival and poor response to chemotherapy.	(36)
SNHG5	Down	Low level of SNHG5 predicts poorer survival.	(155)

### Long Non-Coding RNAs as Therapeutic Targets

LncRNAs play crucial roles in practically all aspects of GC progression, including drug resistance, suggesting their great clinical value as therapeutic targets or therapeutic agents in GC patients, particularly those exhibiting a poor response to chemotherapy. Targeting these lncRNAs may provide novel insights into the development of therapeutic strategies for reversing GC drug resistance. For instance, Xu et al. showed that lncRNA DANCR was strongly upregulated in CDDP-resistant GC tissues and cell lines, and its knockdown by small interfering RNA (siRNA) was shown to decrease survival and increase apoptosis in CDDP-resistant GC cells (52). Lan et al. revealed that lncRNA antisense non-coding RNA in the INK4 locus (ANRIL) was highly expressed in CDDP and 5-FU-resistant GC tissues and cell lines. The knockdown of ANRIL by siRNA could inhibit the development of the MDR of GC cells to CDDP and 5-FU by upregulating the expression of *MDR1* and *MRP1* (34). Han et al. found that the high expression of lncRNA X inactive-specific transcript (XIST) contributed to the CDDP resistance of GC cells. The knockdown of XIST by siRNA significantly inhibited cell proliferation and promoted apoptosis *via* modulating the expression of Aurora kinase B (*AURKB*) (50). These evidences strongly suggest that lncRNAs possess great potential as effective therapeutic targets reversing GC drug resistance. Thus, the identification and synthesis of novel drugs targeting lncRNA is a promising pathway for the clinical treatment of GC patients, particularly those with drug resistance. Furthermore, lncRNAs can be regulate by several oncogenic signaling pathways, such as Wnt/β−catenin and STAT3 signaling pathways (157, 158), indicating that the screening and synthesis of novel inhibitors targeting the nodes of these pathways is also an attractive strategy to treat GC. In addition, several efficient RNA‐based techniques, such as siRNA, clustered regularly interspaced short palindromic repeats (CRISPR)/CRISPR-associated (Cas) protein 9 (CRISPR/Cas9), epigenetic modifiers, and transcription activator-like effector nuclease, are also used to target lncRNAs in GC treatment (159). However, lncRNA-based therapies have not yet been translated into GC treatment in clinic, and further investigations are still needed to elucidate the mechanisms of lncRNAs in GC drug resistance.

## Conclusion and Perspective

GC is the most common malignant diseases in the digestive system, with high recurrence and mortality rate (139). Chemotherapy remains the first-line standard therapeutic method for GC patients and has efficiently improved patients’ prognosis. However, most patients eventually develop resistance to chemotherapeutic agents, leading to the failure of clinical treatment and, ultimately patients’ death (160). In recent years, with the rapid development of high-throughput sequencing techniques and bioinformatics, a large number of aberrantly expressed lncRNAs were found to be involved in GC drug resistance *via* various mechanisms, including targeting the oncogenic signaling pathways, inhibition of apoptosis, induction of autophagy, modulation of CSCs, and promotion of the EMT process.

**Figure 5:**
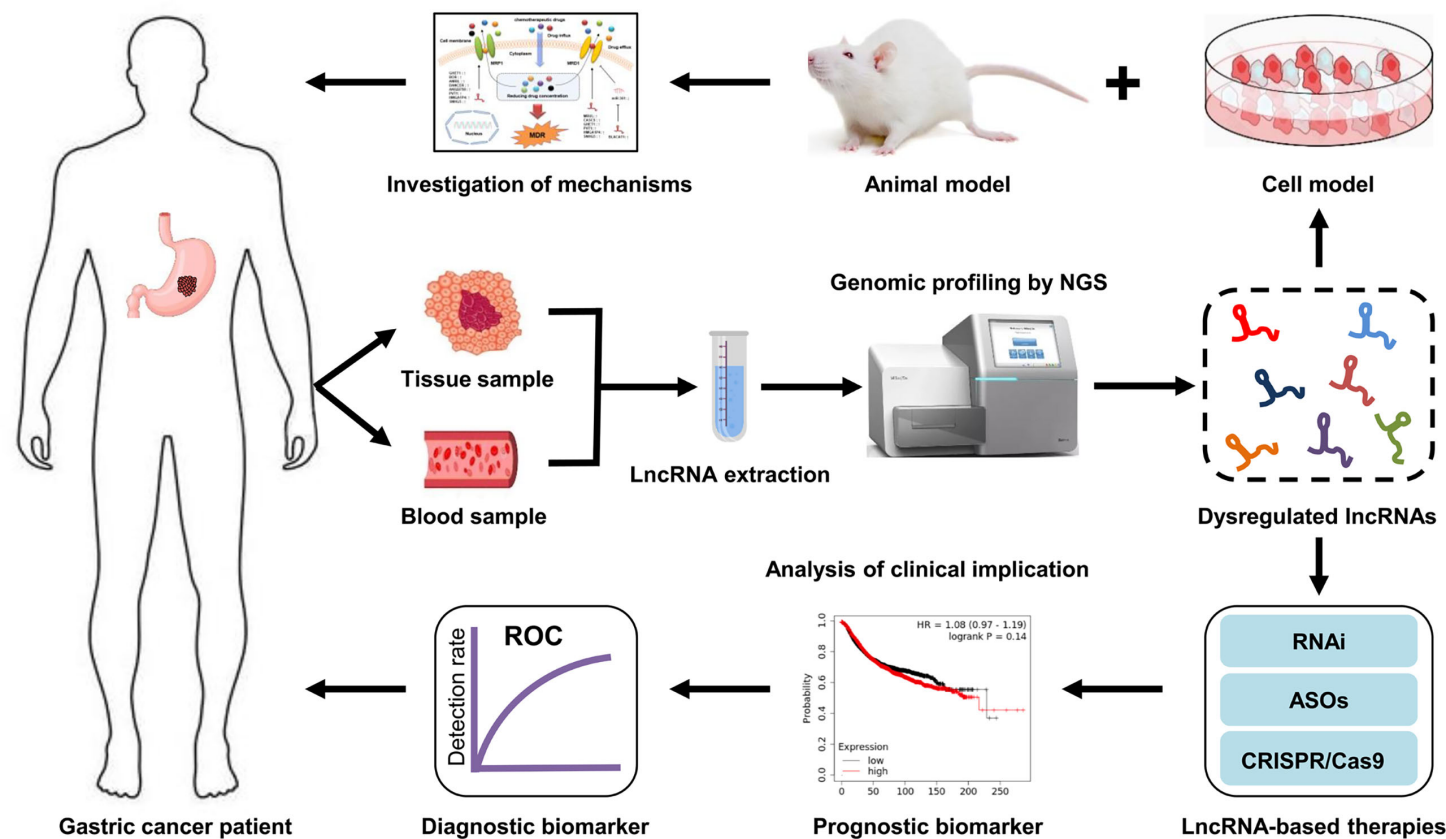
Clinical implications of lncRNAs in GC drug resistance. LncRNAs are extracted from tissue or blood samples of GC patients. Next, the expression profiles of lncRNA are analyzed by high-throughput sequencing technologies. Subsequently, the dysregulated lncRNAs are screened *via* bioinformatics analysis. The mechanisms of drug resistance-related lncRNAs in GC are investigated using cell and animal models. The aberrantly expressed lncRNAs that can serve as diagnostic or prognostic biomarkers for drug resistant-GC patients are identified. Additionally, targeting specific lncRNAs may be a new therapeutic strategy for GC patients, particularly those with a poor response to chemotherapy.

Early diagnosis and prognostic assessment are crucial for the effective treatment of GC patients (161). Therefore, the screening and identification of valuable biomarkers with a high specificity and sensitivity is of great importance in the development of individual diagnose and therapeutic strategies for GC patients, particularly those with a poor response to chemotherapy. Accumulating studies suggest that lncRNA profiling can provide a large number of valuable information for cancer diagnosis, prognosis and treatment. This means that it is plausible that integrating lncRNA data into clinical trials to confirm their clinical implication. Based on the association observed between lncRNA and GC progression, it is possible to assume that specific lncRNAs will shortly be used as clinical biomarkers for the diagnosis and prognosis of GC patients. In addition, lncRNAs are also recognized as therapeutic targets or therapeutic agents for GC patients (21). Compared with protein-coding genes, lncRNA therapy exhibits several advantages: Firstly, lncRNAs are highly abundantly expressed in humans with about 5,400 to more than 10,000 lncRNA transcripts (162), indicating that lncRNAs could be more accessible targets for cancer therapy. Secondly, there are no drug resistance reports for lncRNA therapy up to now. Thirdly, chemical modifications can significantly enhance the activity and stability of lncRNAs (163). Currently, some molecular techniques based on lncRNAs, such as siRNA, CRISPR/Cas9, epigenetic modifiers, and transcription activator-like effector nuclease, have been applied to the treatment of cancers, including GC (**Figure 5**). However, some limitations, such as side effects, off-target effects, and a lack of a reliable delivery system, still exist in translating these research findings on lncRNA-mediated GC drug resistance into clinical application. Nevertheless, lncRNA-based therapeutic strategies have exhibited promising potential in helping GC patients overcome drug resistance. However, in-depth investigations are further required to elucidate the exact mechanisms of lncRNAs in GC drug resistance.

In summary, research on the association between lncRNAs and GC drug resistance has attracted much attention, with an increasing number of lncRNAs proved to be involved in GC drug resistance. However, our overall understanding of this relationship is still insufficient, and further studies are required to fully elucidate their relevant mechanism, and these may provide novel insights into the development of therapeutic strategies to reverse GC drug resistance.

## Author Contributions

YL: Writing—conceptualization, original draft preparation; writing—review and editing, funding acquisition. XA: Data curation, writing—review and editing. YW: Data curation. XL: Data curation. JW: Writing—review and editing. All authors contributed to the article and approved the submitted version.

## Funding

All authors are supported by Qingdao Medical College, Qingdao University. This work was funded by the National Natural Science Foundation of China (81802822) and the China Postdoctoral Science Foundation (2018M642607).

## Conflict of Interest

The authors declare that the research was conducted in the absence of any commercial or financial relationships that could be construed as a potential conflict of interest.

## Publisher’s Note

All claims expressed in this article are solely those of the authors and do not necessarily represent those of their affiliated organizations, or those of the publisher, the editors and the reviewers. Any product that may be evaluated in this article, or claim that may be made by its manufacturer, is not guaranteed or endorsed by the publisher.
